# Comparison of knowledge of occupational hazards of lead exposure and blood lead estimation among roadside and organized panel beaters in Enugu metropolis, Nigeria

**DOI:** 10.11604/pamj.2021.40.47.28281

**Published:** 2021-09-17

**Authors:** Chukwukasi Wilson Kassy, Ndubuisi Casmir Ochie, Ifeoma Juliet Ogugua, Chidozie Reginald Aniemenam, Chikee Elias Aniwada, Emmanuel Nwabueze Aguwa

**Affiliations:** 1 Department of Community Medicine, University of Nigeria Teaching Hospital, Enugu, Nigeria; 2 Department of Community Medicine, Nnamdi Azikiwe University Teaching Hospital, Nnewi, Nigeria,; 3 Department of Community Medicine, University of Nigeria, Nsukka, Enugu Campus, Enugu, Nigeria

**Keywords:** Lead poisoning, knowledge, risk factors, informal sector, Nigeria

## Abstract

**Introduction:**

occupational practices continuously exposes workers to hazards of lead. This study aimed to compare the knowledge of occupational hazards associated with lead exposure, and blood lead estimation among roadside and organized panel beaters in Enugu metropolis, Nigeria.

**Methods:**

this was a cross-sectional study. Multistage sampling method was used to select 428 panel beaters in Enugu metropolis. Samples were analyzed using Atomic Absorption Spectrometer at 283.3 wavelengths. Data were entered and analyzed using Statistical Package for Social Science 20. Comparative analysis were done using chi - square, T-test, Mann-Whitney U-test, Kruskal-Wallis test, logistic regression and level of significance was set at 5%.

**Results:**

the majority of respondents on both roadside (59.8%) and organized (73.4%) sectors had poor knowledge of hazards of lead exposure. The difference was significant using χ^2^ (P<0.05). The median blood lead levels were 3.0µg|dl and 16.0µg|dl for roadside and organized panel beaters respectively. The difference was significant with Mann-Whitney U test (P<0.001). The prevalence of elevated blood lead at 10µg|dl were 36.9% (roadside sector) and 64.5% (organized sector). The duration of working hours (OR = 4.34, CI = 1.729 – 10.338) was found to be the predictor of elevated blood lead levels.

**Conclusion:**

there were general poor knowledge of hazards of lead exposure and high prevalence of elevated lead levels which were more among organized panel beaters. Advocacy on standard organizational structures that support improved occupational health practices is needed and routine outreach by research institutions for health education and safety training.

## Introduction

Occupational lead poisoning is a known health hazard for many years, from 370 B.C when Hippocrates attributed severe cases of abdominal colic to lead (Pb) [1]. Nicander of Colophon in the 2^nd^ century B.C associated pallor, colic, paralysis and drooping limbs with Pb exposures [2]. Pliny the elder, (A.D 23 - 79) wrote that workers painting ships with white Pb wore loose bags over their faces for prevention [1]. In the Middle Ages, Stockhausen, attributed miner´s asthma to the Litharge [2, 3]. Bernardino Ramazzini (1633- 1714) noted that workers health and their illness should be studied because of the dangers of working with Pb [2]. By the 19^th^ and 20^th^ centuries, industrialization had caused epidemics of Pb poisoning with toxicity in high doses, both acute and clinically overt [3, 4]. These were controlled through the Legge´s aphorism and subsequently by restriction on the use of tetraethyl lead in gasoline [3, 5, 6].

Panel beaters are part of automobile technicians whose occupational practices of panel beating, repairs, cuttings, soldering, welding and spray paintings expose them to Pb poisoning [7]. The organized panel beaters are formal sector workers under controlled occupational safety practices, factories act, labour laws and workman compensation act, unlike the roadside panel beaters who are informal sector workers [8]. The proliferation of transport businesses and increase cost of cars in Enugu State and Nigeria has increased the need for repairs and consequent exposure to occupational Pb poisoning. Pb occurs in form of inorganic and organic lead and enters the body through inhalation of lead dust and fumes, ingestion through the mouth and absorption by the skin [1, 9-11]. The consequent health effects are from respective target organs causing haematologic, neurologic, nephro, cardiovascular and reproductive toxicities [5]. These were found previously at acute and high dose exposures to Pb, however, current evidence indicates toxicities at chronic and low dose exposures of less than 10µg|dl [12]. The exposures are best estimated and monitored using biomarkers of which whole blood is the primary biomarker and the most reliable index [13, 14].

The prevalence of Pb poisoning has been estimated over years at varying thresholds from 60µg|dl to currently to less than 10µg|dl for adult workers and 5µg|dl for children [9, 12-14]. This implies that there is no safe level of exposure to Pb and as the Pb levels increases, the severity of damage to target organs increases [15]. Pb poisoning account for 0.9% of the total global diseases resulting in 13.9 million Disability Adjusted Life Years (DALYs) worldwide and 540,000 deaths [1, 16, 17]. High burden of this disease occur in developing countries of sub-Saharan Africa and South East Asia [16]. Institute for Health Metrics and Evaluation estimated in 2016 that Pb exposure accounted for 63.8% of global idiopathic developmental intellectual disability, 3% of Ischaemic heart disease and 3.1% of stroke with highest burden more in low and medium income countries [17]. The knowledge of hazards of Pb exposure are poor. Reports from South Africa showed 11% level of knowledge and Ohio U.S.A showed less than 50% knowledge of Pb poisoning [18-20]. The prevalence of Pb are still high, study in Jamaica found that at blood lead (BPb) of 60µg|dl, the prevalence among battery shop workers was 65% while the prevalence among battery manufacturing workers was 28% [21]. Study in Lagos, Nigeria, found that at 40µg|dl, prevalence among organized sector automobiles workers was 40.3% while the prevalence among roadside automobile workers was 34.3% [1].

Comparing the two sectors of panel beaters will help to unearth the institutional and organizational gaps that exposes panel beaters to hazards of Pb and provide information for a comprehensive Pb poisoning prevention strategy. Estimating BPb levels at lower threshold of less than 10µg|dl will also help understand the burden of low dose chronic exposures to Pb and the potentials of subclinical Pb poisoning. Ultimately, the study will help workers understand the various practices in their work that influenced or promoted Pb exposures like frequency of work, length of working hours, years of experience and other inhibitory factors which were easily neglected by workers like use of personal protective equipment (PPE) and maintaining occupational safety and hygiene. This study aimed to compare the knowledge of occupational hazards associated with Pb exposure, estimate BPb levels and determine factors associated with knowledge of hazards of Pb exposure and the BPb levels among roadside and organized panel beaters in Enugu metropolis, Nigeria.

## Methods

**Study design and site:** this was a cross-sectional analytical study of roadside and organized panel beaters in Enugu metropolis. Enugu metropolis is the capital of Enugu State in the south east geo - political zone of Nigeria, from where it derives its name. The metropolis is constituted by three Local Government Areas in Enugu State which are Enugu North, Enugu South and Enugu East and is inhabited mostly by the Igbo ethnic group. According to the 2006 National Population census, the Local Government Areas have a population of 722,664 [22, 23], which is 22.2% of Enugu State population and 0.51% of Nigerian total population. The metropolis is an administrative and educational town predominated economically by public services, trading and transportation [24]. The panel beaters in this part of the state do not have centralized garage i.e. mechanic village. They concentrate mainly on the streets, along the roads and very close to vehicle spare parts markets. They are registered under several trade but autonomous unions with respect to their locations. The organized panel beaters are privately owned by the transport companies that commute within and outside the state transporting people to other states in the country.

**Sample size and selection:** the study population were panel beaters and trainees who had spent over one year and are willing to participate in the study while those on chelation therapy, on allopurinol, severely ill or with chronic diseases were excluded. The minimum sample size for the study was determined using the formula for comparing two independent proportions and 14.3% representing the proportion of Nigerian adults with BPb levels >20µg|dl [25, 26].

A minimum sample size of 214 per each sector was obtained after correcting for non-response rate. Multistage sampling technique was used for both roadside and organized panel beaters in this study. For the roadside panel beaters, the first stage was a selection of Enugu North among the three local government areas by simple random sampling as a zone using balloting method. Second was selection of one division out of the 5 divisions in Enugu North LGA by simple random sampling using balloting method. Third was selection of 10 branches out of the 13 branches in Enugu North LGA by simple random sampling using balloting method. Lastly, stratification and proportionate allocation of panel beaters from all the workshops within the selected branches was done. Based on the density of workers, workshops concentration and proximity to the central motor spare parts and scraps market. The workshops were further classified into small (<5 panel beaters), medium (5-10 panel beaters) and large workshops (>10 panel beaters) with 29, 16 and 9 workshops respectively out total of 54 workshop, using proportionate allocation a total of 228 panel beaters [2X29] + [5X16] + [10X9] were selected. For the organized panel beaters, first was the selection of Enugu North among the three local government areas by simple random sampling. Second was proportionate allocation of government and private owned company workers using ratio of 1: 3. 56 panel beaters were selected out of 70 panel beaters by simple random sampling using balloting method. The private owned workshops were categorized into small (2 - 4 panel beaters), medium (5 - 7 panel beaters) and large (8 - 12 panel beaters) workshops. There were a total of 10 small, 16 medium and 9 large workshops. 170 panel beaters [2X10] + [5X16] + [10X9] were selected. A total of 226 panel beaters were selected from the organized panel beaters taking into consideration missed and incomplete responses.

**Community entry and advocacy:** letters of intent were written to the secretary of panel beaters of Enugu North Local Government, the Executive Secretary of the government owned transport companies and the various managers of the privately owned companies. Attached to the letters were summary of the proposals and ethical clearance. Appointments were secured and further meetings were held with each of the branches at their monthly meetings where lectures were given on the reasons, benefits and conduct of the research. The same was done for organized panel beaters involving the chairman/managers, and their foremen, after approvals were gotten from their headquarters. Finally, date was given for the actually conduct of the research. The results of the research were relayed back to respondents including health education and recommendation made to the heads of the workshops.

**Data and sample collection:** interviewer administered semi structured questionnaire was used to collect data from the roadside and organized panel beaters from November 2018 - February 2019. The questionnaire was adapted from the medical evaluation questionnaire for occupational Pb exposure by the Massachusetts division of occupational safety [27]. The questionnaire was pre-tested in Enugu East. About 20 samples were collected from both sectors for pretest. The observed shortcomings in relevance and scope of questions was corrected before final administration of the questionnaires to the respondents. Data and samples were collected using research assistants who were three resident doctors and three phlebotomists. They were trained for two days, two hours per day on sample collection procedures and questionnaire administration. Also included were good communication and follow up skills, objectives of the study and ethical issues that were involved in the research. BPb were collected under aseptic procedure and were analyzed at Project Development Agency (PRODA) Enugu. Blood sample collection was done in an enclosed well screened place. The venipuncture system was used to perform a venipuncture and the desired blood of about 2 - 3mls were drawn into EDTA vacutainer bottle for BPb estimation. The samples were transported immediately to the laboratories using the Gio style cold box after each day, accompanied by 5 and 10mls syringes, bleach and gloves for maintenance of universal precautions. The blood samples were diluted to 10mls using deionized water because of accompanying cations and anions. The diluted sample were aspirated by the Atomic Absorption Spectrometer (AAS) via a capillary tube at a wavelength of 283.3nm for lead analysis.

**Statistical analysis:** data were entered and analyzed using Statistical Package for Social Science version 20. Exposure variables contained in the questionnaires were socio-demographic variables, risk factor variables as working hours, frequency of work and work experience. Outcome variables were knowledge of occupational hazards of lead exposure and BPb levels. Categorical variables were summarized using frequency tables and proportion while continuous variables were summarized using mean, standard deviation, medium and inter-quartile range. Twenty five variables were used to assess knowledge of occupational hazards associated with Pb exposure. The questions were Yes or No, the wrong answer were awarded zero score and the correct answer were awarded a score of 1. The scores were summed up into a knowledge score of 25. Those with scores of 50% and above were noted as good knowledge while those with scores less than 50% were noted as poor knowledge [28]. The proportion of respondents with good knowledge were compared using chi square test of statistical significance. The Pb levels in blood were categorized using cut off of <10µg|dl, 10-40µg|dl and >40µg|dl for unexposed, acceptable and permissible/dangerous levels respectively [29]. Prevalence of Pb poisoning were noted as proportion of panel beaters with BPb of 10µg|dl and above. Mean, standard deviation, median, interquartile range for skewed data were used for summary analysis. Comparison of variables were managed using chi-square, T-test, Mann-Whitney U-test (MHU) and Kruskallis Wallis test (KWT). Logistic regression was used to determine predictors and confounders of elevated blood lead levels. Ninety-five percent (95%) confidence interval were used for level of significance.

**Ethical considerations:** ethical approval was obtained from the Health Research Ethics Committee of the University of Nigeria Teaching Hospital Ituku/Ozalla, Enugu. Permission was obtained from unions of panel beaters and organized panel beaters in Enugu State. A written informed consent was obtained from participants.

## Results

**Socio-demographic characteristics:** a total of 428 participants were studied. The mean ages (SD) were 31.1 ± 10.3 years and 37.9 ± 12.1 years for roadside and organized panel beaters respectively. All the respondents on both sectors are male, of Igbo ethnic group and majority are of Christian religion. Majority, 70.6% and 56.5% of the respondents from the roadside and organized sector respectively had secondary education. Many, 59.8% of the roadside panel beaters were single compared to 36.9% of organized panel beaters. Most, 72.9% of roadside workers earn more than their counterpart, 50% in organized sector.

**Knowledge of occupational hazards of lead exposure:** less than half, 42.5% and less than one - third, 26.6% of the roadside and organized panel beaters respectively have heard of Pb. Only 35.0% and 22.9% of roadside and organized panel beaters respectively knew that Pb is poisonous. Less than half of respondents knew of various sources of Pb, factors that increase exposure to lead, route of entry and preventive measure to Pb exposure. The general knowledge of respondents were poor. Little above half, 59.8% of roadside panel beaters had poor knowledge while 73.4% of the organized panel beaters had poor knowledge. The difference in knowledge was significant (P < 0.05) (Table 1).

**Table 1:** knowledge of occupational hazards associated with lead exposure

Variable	Roadside panel beaters. N = 214		Organized panel beaters. N = 214		Statistical analysis N = 428	
	Frequency	Percent	Frequency	Percent	χ2	P value
Awareness of lead	91	42.5	57	26.6	11.94	0.001*
Lead is poisonous	75	35.0	49	22.9	7.675	0.006*
**Sources of Lead**						
Metal	76	35.5	57	26.6	3.938	0.047*
Alloyed Lead	73	34.1	57	26.6		
Paints	73	34.1	57	26.6		
Glass	45	21.0	57	26.6		
Car batteries	73	34.1	57	26.6		
Petrol	71	33.2	57	26.6		
**Work practices that exposes to Lead**						
Panel beating	79	36.9	57	26.6	5.216	0.022*
Cutting metal	70	32.7	57	26.6		
Soldering	71	33.2	57	26.6		
Welding	74	36.6	57	26.6		
Spray/painting	78	36.4	56	26.3		
**Increase exposure to Lead**						
Longer duration	88	41.1	57	26.6	10.023	0.002*
Frequency of job	86	40.2	57	26.6		
Years of practice	88	41.1	56	26.2		
**Route of entry**						
Inhalation	87	40.7	57	26.6	9.419	0.002*
Ingestion	84	39.3	57	26.6		
Skin contact	86	40.2	57	26.6		
Body system	88	41.2	57	26.6		
**Preventive measures**						
Training and education	87	40.7	57	26.6	9.419	0.002*
Personal hygiene	89	41.6	57	26.6		
Workplace hygiene	86	40.2	57	26.6		
Personal Protective equipments	91	42.5	57	26.6		
Pre–employment/routine exam	66	30.8	57	26.6		
**Overall Knowledge**						
**Poor knowledge**	**128**	**59.8**	**157**	**73.4**	**8.832**	0.03*
**Good knowledge**	**86**	**40.2**	**57**	**26.6**		

* Significant

**Blood lead estimation:** the median BPb level among roadside panel beaters was 3.0µg|dl and inter - quartile range of 0 - 20µg|dl compared with organized panel beaters of 16.0µg|dl and inter - quartile range of 4-31.3µg|dl. The prevalence of elevated BPb levels among roadside and organized panel beaters at ≥10µg|dl were 36.9% and 64.5% respectively. The differences were significant (P < 0.0001) (Table 2).

**Table 2:** lead estimation of roadside and organized panel beaters

	Roadside panel beaters N = 214		Organized panel beaters N = 214		Statistical analysis N = 428	
Variables	Frequency	Percent	Frequency	Percent		
**Blood lead**					**MHU**	P value
Median	3.0µg|dl		16.0µg|dl		-2.720	<0.0001*
Inter–quartile range	0–20µg|dl		4-31.3µg|dl			
**Thresholds categories**					**Chi-square**	
<10µg|dl (unexposed/normal)	135	63.1	76	35.5		
10–40µg|dl (acceptable)	55	25.7	105	49.1	33.54	<0.0001*
>40µg|dl (permissible/dangerous)	24	11.2	33	15.4		

*Significant

**Association of knowledge level and blood lead:** among the roadside panel beaters, 55.6% of those with unexposed/normal BPb levels, 63.6% of those with acceptable BPb levels and 75.0% of those with permissible and dangerous BPb levels had poor knowledge. While among the organized panel beaters, 76.3% of those with unexposed/normal BPb levels, 72.4% of those with acceptable blood lead levels and 69.7% of those with dangerous BPb levels had poor knowledge. The association was neither significant in both sectors (Table 3).

**Table 3:** association between knowledge of hazards of lead exposure and blood levels of lead among roadside and organized panel beaters

Variable	Overall knowledge		Statistical analysis	
	Poor	Good	χ2	P value
**Blood lead**				
**Roadside**				
<10µg|dl (unexposed/normal)	75 (55.6)	60 (44.4)	3.655	0.161
10–40µg|dl (acceptable)	35 (63.6)	20 (36.4)		
>40µg|dl (permissible and dangerous)	18 (75.0)	6 (25.0)		
**Organized**				
<10µg|dl (unexposed/normal)	58 (76.3)	18 (23.7)	0.618	0.734
10–40µg|dl (acceptable)	76 (72.4)	29 (27.6)		
>40µg|dl (permissible and dangerous)	23 (69.7)	10 (30.3)		

**Factors associated with knowledge of hazards of lead exposure:** among the roadside panel beaters, age (χ^2^ = 7.333, P = 0.026), educational level (χ^2^ = 8.115, P = 0.017) and marital status (χ^2^ = 10.583, P = 0.001) were found to be associated with knowledge of hazards of Pb exposure. For the organized panel beaters, age (χ^2^ = 19.403, P < 0.001) and educational level (χ^2^ = 10.058, P = 0.007) were associated with hazards of Pb exposure (Table 4).

**Table 4:** factors associated with knowledge of hazards of lead exposure among roadside and organized panel beaters

Variable	Poor knowledge	Good knowledge	Chi - square	P value
**Roadside**				
**Age (years)**				
16 – 30	82 (67.8)	39 (32.2)	7.333	0.026*
31 – 50	40 (49.4)	41 (50.6)		
51 – 80	6 (50.0)	6 ( 50.0)		
**Education**				
Primary and none	32 (58.2)	23 (41.8)	8.115	0.017*
Secondary	95 (62.9)	56 (37.1)		
Tertiary	1 (12.5)	7 (87.5)		
**Marital status**				
Single	88 (68.8)	40 (31.2)	10.583	0.001*
Married	40 (46.5)	46 (53.5)		
**Monthly income**				
<35000	40 (69.0)	18 (31.0)	2.773	0.960
>35000	88 (56.4)	68 (43.6)		
**Organized**				
**Age**				
16 – 30	63 (86.3)	10 (13.7)	19.403	<0.001*
31 – 50	76 (73.8)	27 (26.2)		
51 – 80	18 (47.4)	20 ( 52.6)		
**Education**				
Primary and none	51 (77.3)	15 (22.7)	10.058	0.007*
Secondary	93 (76.9)	28 (23.1)		
Tertiary	13 (48.1)	14 (51.9)		
**Marital status**				
Single	64 (81.0)	15 (19.0)	3.749	0.053
Married	93 (68.9)	42 (31.1)		
**Monthly income**				
<35000	74 (69.2)	33 (30.8)	1.937	0.164
>35000	83 (77.6)	24 (22.4)		

* Significant.

**Factors associated with blood lead levels:** among the roadside panel beaters, the frequency of work (P < 0.0001) was found to be associated with Pb poisoning while among the organized panel beaters, the duration of working hours (P < 0.0001) and years of working experience (P < 0.04) were associated with Pb poisoning. The duration of working hours (OR = 4.34, CI = 1.729 - 10.338) was found to be the predictor of elevated BPb levels among the organized panel beaters (Table 5, Table 6).

**Table 5:** factors associated with blood lead level among roadside and organized panel beaters

	ROADSIDE				ORGANIZED			
Variable	Freq	Mean Rank	Stat. Test	P value	Freq	Mean Rank	Stat. Test	P value
**Age**			**KWT**				**KWT**	
16 – 30	121	113.23		0.258	73	99.20		0.353
31 – 50	81	98.97			103	110.88		
51 – 80	12	107.29			38	114.28		
**Education**								
Primary and none	55	106.64		0.962	66	106.34		0.767
Secondary	151	108.07			121	106.33		
Tertiary	8	102.63			27	115.59		
**Marital status**			**MHU**				**MHU**	
Single	128	111.63		0.222	79	104.34		0.567
Married	86	101.35			135	109.35		
**Monthly income**								
<35000	58	103.45		0.550	107	108.41		0.829
>35000	156	109.01			107	106.59		
**Working hours**			**MHU**				**MHU**	
1 – 8	15	104.00		0.816	47	133.44		0.001*
9 – 14	199	107.76			167	100.20		
**Frequency of work**								
2 -3 times per week	168	85.32		<0.0001*	0	0.00	**Not applicable**	
>3 times per week	46	188.49			214	107.50		
**Years of experience**			**KWT**				**KWT**	
1 – 10 years	125	108.72		0.886	105	101.05		0.04*
11 – 20 years	56	107.49			57	125.26		
21 – 50 years	33	102.91			52	101.05		

* Significant

**Table 6:** predictors of lead poisoning among panel beaters

Variables	Odds ratio (Exp B)	Lower C.I	Upper C.I	Significance
**Roadside panel beaters**				
**Working hours**				
1 – 8	0			
9 – 14	1.24	0.319	4.817	0.757
**Frequency of work**				
2 – 3 times per week	0			
>3 times per week	0.0	0.000	-	0.997
**Years of experience**				
1 – 10 years	0			
11 – 20 years	1.72	0.538	5.472	0.362
21 – 50 years	0.99	0.250	3.88	0.984
**Organized panel beaters**				
**Working hours**				
1 – 8	0			
9 – 14	4.23	1.729	10.338	0.002
**Years of experience**				
1 – 10 years	0			
11 – 20 years	0.913	0.433	1.926	0.811
21 – 50 years	2.091	0.864	5.057	0.102

## Discussion

The overall knowledge of occupational hazards of Pb exposure was found to be poor for both sectors of panel beaters with significant difference between them. This agreed with study in South Africa, Chicago, Illinois and Ohio, USA [18-20]. The reason could be because automobile repairers are mostly unskilled labourers without formal designated curriculum for transfer of knowledge. The knowledge acquired are by accidental transfer of knowledge by researchers from institutions of learning or through self-learning, observation and master apprentice transfer of knowledge [30, 31]. The restrictiveness of the organized sector further explain the poorer knowledge among her workers. This can also partly be explained by the fact that workers are not made known of all the hazards they work with against the principles postulated in Legge´s aphorism. The study in Lagos, Nigeria reported high level of awareness among respondents on Pb hazards on both sectors [1]. However, this study did not look into other knowledge questions like sources of Pb, work practices that exposes to Pb, route of entry, duration of exposure and preventive measures to Pb hazards. This was further buttressed by study in Benin, Nigeria that found that level of awareness of occupational hazards does not commensurate with preventive measures [32].

The BPb is commonly measured as a primary biomarker showing the body burden and absorbed doses of Pb [13, 14]. The Occupational Safety and Health Administration (OSHA) unit of United States Department of Labour interprets Pb levels in adult workers as <10µg|dl to be unexposed or normal; 10 - 40µg|dl to be acceptable levels for long term exposure and possible retest in 6 months and >40µg|dl as permissible and dangerous levels for medical removal and retest within 1 month until blood is <40µg|dl [29]. The current study found that the median BPb levels were 3.0ug|dl and 16.0µg|dl for roadside and organized panel beaters respectively which were not comparable. The prevalence of BPb among roadside sector was 36.9% while that among organized sector 64.5%. This showed that, there is generally higher levels of Pb among organized panel beaters averagely within the acceptable range. They operate in a closed environment that limit health education and safety training intervention program thereby increasing exposure to hazards of lead unless the organization has an occupational health physician or safety engineer. This agreed with study in Lagos, Nigeria, where both sectors were compared [1].

Other studies on roadside sector workers done in Nnewi, Nigeria, Jua Kali, Kenya and Jimma, Ethiopia reported similar BPb and prevalence [33-35] Other studies on organized sectors done among boatyards workers in southern Thailand, battery manufacturing plant in Thailand and in South Korea found high BPb and high prevalence of Pb [36-38]. The similarities of this studies showed that chronic exposures to Pb continues to be of public health concerns among occupational groups even at low thresholds of 10µg|dl. The weak or absent occupational health practices mostly in low or medium income countries coupled with poor knowledge and training in health and safety measures against hazards of occupational health exposures compounds the conditions of these workers. The higher Pb levels encountered in organized sectors could be better explained by their frequent exposures where 100% work daily (>3 times per week) compared to infrequent exposures among roadside sectors where majority work 2-3 times per week. The craftsmen in the roadside sectors are their own determinant of the frequency of work exposure. They do have interval free periods with exposure to work disrupting the steady state required for high Pb burden unless incases of outbreaks (epidemic focus) as was seen in Zamfara Nigeria among artisanal gold miners and Dakar Senegal among battery repairers [39, 40]. Other reasons for higher Pb levels among organized panel beaters include the observed enclosed or warehouse like workshops with ineffective local ventilation control system compared to the open spaces that offer greater dilution to atmospheric Pb as well as inefficient administrative and engineering control measures as observed in study done in Lagos [1]. Further studies using qualitative assessment among the administrative actors to identify other differences is advocated.

The knowledge of hazards of Pb exposure was found in this study not to be associated with BPb levels. Knowledge on occupational hazards helps in hazards identification, sources of hazards and various preventive measures. However, exposure to Pb poisoning or other poisoning agents could be due to the actual practices within the work processes or job. Interventions of health education to improve knowledge base of Pb poisoning should concurrently be accompanied by intervention on safety training. This possibly will close any knowledge - practice gap that exposes panel beaters to increase lead exposure and consequent Pb poisoning.

Age, education and marital status were found to have a significant association on knowledge among roadside panel beaters while only age and education was found to be significantly associated among organized panel beaters. The above factors agreed with study in Chicago and China [19, 41]. This is because age and education helps to increase knowledge of occupational hazards. The more advanced and educated workers are most likely to be experienced through repeated exposure to occupational environment, materials, practices and processes.

The association between socio - economic factors (age, educational level, marital status and monthly income) and Pb levels among panel beaters of both sectors were found not to be significantly associated with BPb levels. This result agreed with study in Cuba [42] but differed with study in Thailand among boatyard workers where association of age and education was found to be statistically significant with BPb [36]. This difference could be due to other factors influencing BPb level like health safety and preventive measures which is totally weak or absent in medium scale organized factory in low income countries like Nigeria that are privately owned. This inferred that preventive measures should be instituted irrespective of age, educational level, marital status and income levels.

Among roadside panel beaters, frequency of work was found to be significantly associated with BPb levels. Among organized panel beaters, duration of working hours and years of work experience were found to be significantly associated with Pb levels. However, duration of working hours was found to be the predictor of elevated BPb levels among the organized panel beaters. Those who spent more than 8 hours per day are 4.23 times more likely to have higher BPb levels compared to those that spent 8 hours and less. This agreed with studies in Lagos, Nigeria, Thailand and Iran [1, 43, 44]. This is because increased working hours leads to constant exposure to Pb and consequent hazardous effects. Frequent exposures also cause increasing higher thresholds (time weighted average exposure of 8 hours for 5 days will be increased) among workers which in turn ensures steady state of chronic low dose exposure. The working hours showed that higher time put in work influences Pb toxicity because it increases the exposure time to lead. The frequency of work among organized sector has fixed time schedule to work because availability of work is sure. Owners of the transport business wants their buses up and doing at any time to commute people. Unlike in roadside, availability of work is sometimes and sparingly in some cases. This could be the reason behind the removal of workers at high BPb levels of >60µg|dl in the past. From this study, despite majority on both sectors working more than 8 hours per day, all panel beaters in the organized sectors worked more than three times per week compared to the roadside panel beaters which work 2 - 3 times per week. This could account for increased exposure and consequent Pb levels among organized panel beaters. The need to control exposure to Pb using time weighted average of 8 hours for 5 days per week is strongly advocated coupled with other administrative and engineering control measures.

**Limitations:** the study is specific to one area and the findings cannot be generalized. Also as a cross-sectional study, the outcomes are limited to the period of study.

### Recommendations

**Government level:** existing factory acts should be strengthen by ensuring that factory inspectorates enforces the factory laws. As a policy, factories inspectorates should ensure that routine biological and environmental monitoring are done. Records of routine Pb monitoring will serve as evidence base for healthy policy and research on practical preventive measures for control of Pb poisoning. Also included would be awareness campaign at national, state and local levels to provide adequate knowledge on the hazards associated with lead exposure, factors associated with Pb poisoning and preventive measures. This will help to improve the knowledge practice gaps that leads to Pb poisoning on both sectors.

**Organizations | institutions | individual level:** advocacy to transport businesses, unions of panel beaters by occupational health physicians from government and institution of learning on the importance of implementation of occupational health practices, labour acts and laws. The occupational health practices should involve routine health education and safety training. The organized sectors will be made to understand the importance of occupational health physicians or have linkages to institutions of learning. The roadside panel beaters unions should be encouraged to have linkage with institutions of learning to help in health education and safety training.

## Conclusion

Findings from this study showed general poor knowledge of hazards associated with Pb exposure amongst the two sectors with significant difference. The median BPb levels were 3.0µg|dl and 16.0µg|dl between the roadside and organized panel beaters respectively. The prevalence of BPb were 36.9% and 64.5% between the roadside and organized panel beaters respectively. Duration of working hours was found to be the predictor of elevated BPb levels which is 4.23 times among those who spent more than 8 hours per day. Finally, this study proves wrong the assumption that roadside or informal occupation are more predisposed to occupational hazards and chemicals compared with the organized occupations. This further strengthens the truth of Legge´s aphorism where greater responsibility is placed on employer of labour. This principle guide implementation of occupational practices, law and acts for successful control of workplace hazards on both sectors of occupation.

### What is known about this topic

- Globally, Nigeria and in Enugu, lead is an ubiquitous metal of which occupational exposure causes health hazards;
- There is no safe level to lead exposure

### What this study adds

- There is general poor knowledge of occupational hazards of Pb exposure among panel beaters of both sectors in Enugu metropolis, Nigeria;
- There are high prevalence of Pb exposure more among organized than roadside panel beaters in Enugu metropolis, Nigeria;
- The increased blood Pb levels among the organized panel beaters are determined by increased duration of work. Those who worked more than 8 hours per day were more likely to have increased blood lead levels compared to those who worked less.
